# Mapping the mitochondrial landscape in T2DM: key findings from 2003-2023

**DOI:** 10.3389/fendo.2024.1474232

**Published:** 2024-11-20

**Authors:** Yi Tan, Mingjun Liu, Xinfeng Zhou, Tianjiao Gao, Jinxu Fang, Sixian Wang, Shaotao Chen

**Affiliations:** ^1^ Departments of Acupuncture and Massage, Changchun University of Chinese Medicine, Changchun, Jilin, China; ^2^ The Affliated Hospital of Changchun University of Chinese Medicine, Changchun, Jilin, China

**Keywords:** mitochondria, T2DM, bibliometric analysis, VOSviewer, CiteSpace

## Abstract

**Backgound:**

T2DM, a chronic metabolic disorder, poses a significant threat to global public health. Mitochondria play a crucial role in the pathogenesis of T2DM. This study intends to investigate the correlation between mitochondria and T2DM over the past two decades (2003-2023) through bibliometric analysis. Its objectives are to pinpoint trends, emphasize research priorities, and establish a foundation for future investigations.

**Methods:**

A literature search was conducted using the SCI-E database. All recorded results were downloaded in plain text format for further analysis. The following terms were analyzed using Vosviewer 1.6.18, citespace 6.3r1, bibliometrix in RStudio (v.4.4.1), and Microsoft Excel 2021: country, institution, author, journal, references, and keywords.

**Results:**

From January 1, 2003 to December 31, 2023, a total of 2,732 articles were retrieved. The United States, China, and Italy contributed most of the records. UNIVERSITY OF CALIFORNIA SYSTEM, INSTITUT NATIONAL DE LA SANTE ET DE LA RECHERCHE MEDICAL INSERM, and US DEPARTMENT OF VETERANS AFFAIRS were the top 3 most productive institutions. rocha milagros, victor victor m had the most publications, followed by roden michael, and petersen kf had the most citations together. DIABETES published the most articles on research on this topic, followed by AMERICAN JOURNAL OF PHYSIOLOGY-ENDOCRINOLOGY AND METABOLISM, DIABETOLOGIA. The key points of this topic are the relationship between mitochondria and T2DM, the skeletal muscle mitochondrial changes observed in T2DM, and the impact of mitochondrial dysfunction on T2DM. Over the past five years, particle dynamics, mitochondrial dysfunction, and mechanism research have emerged as significant focal points in this field.

**Conclude:**

This paper successfully identified the key areas and emerging trends in the relationship between mitochondria and T2DM, thereby offering valuable insights for future research.

## 1 Introduction

Diabetes is a chronic metabolic ailment marked by persistently high blood glucose levels. Prolonged exposure to such conditions can cause harm to the heart, blood vessels, eyes, kidneys, and nerves. Type 2 Diabetes Mellitus (T2DM) represents a chronic metabolic disorder (1) that is primarily distinguished by compromised insulin secretion from pancreatic beta cells, coupled with a decreased capacity of insulin-sensitive tissues to respond adequately to insulin (2). In recent years, the prevalence of T2DM has been steadily increasing due to various factors such as obesity, high-calorie diets, and inadequate physical exercise. This trend has caused profound psychological and physical distress for patients and their caregivers, while also posing a significant burden on the healthcare system (3). Increasingly, the effective regulation and control of T2DM has garnered significant attention.

Mitochondria are ancient endosymbiotic organelles containing their own circular double-stranded DNA (mtDNA), which encodes 13 polypeptides (4), These peptides are mainly part of the electron transfer chain (ETC) (5). The mitochondrial ETC is the site of oxidative phosphorylation (OXPHOS) (6), which is key to glucose metabolism and the largest net producer of ATP in mammalian cells. Mitochondria are essential for ensuring energy production in cells. Compelling evidence suggests that T2DM and its complications are associated with mitochondrial structural damage and dysfunction (7). The study highlights that high-fat diets have been blamed not only for peripheral insulin resistance, but also for mitochondrial dysfunction in the heart and brain as well as oxidative stress in rats (8). T2DM have altered mitochondrial morphology and reduced respiratory chain activity. Mitochondria are also essential for insulin secretion from pancreatic β-cells (9). These studies suggest that mitochondria are important players in the pathophysiology of diabetes.

Mitochondria, as the ‘energy factories’ of the cell, are highly dependent on a variety of nutrients for their proper functioning. These nutrients are not only involved in energy-generating processes in mitochondria (e.g., the tricarboxylic acid cycle and the electron transport chain), but also help to protect mitochondria from oxidative stress and promote mitochondrial biosynthesis and self-renewal. For example, coenzyme Q10 is a key component of the mitochondrial electron transport chain (10) and is responsible for transferring electrons in the inner mitochondrial membrane to generate ATP (cellular energy). In addition, coenzyme Q10 has antioxidant properties and reduces reactive oxygen species (ROS) generated during electron transfer. Appropriate intake of coenzyme Q10, B vitamins, magnesium, iron, and antioxidants can maintain the efficiency of mitochondrial energy metabolism, reduce the damage of oxidative stress, and promote mitochondrial biosynthesis.

In T2DM, there may be gender differences in the onset and progression of mitochondrial dysfunction. It usually manifests itself in the following four ways: The effects of sex hormones (11), oestrogen, have been shown to have a protective effect on mitochondrial function, and testosterone levels in men have been implicated in metabolic regulation. Differences in mitochondrial biosynthesis and metabolism (12, 13) Females typically have higher mitochondrial densities and greater mitochondrial function than males, especially in skeletal muscle. Males are generally more efficient than females in glucose metabolism, especially in the uptake and utilisation of glucose in muscle. Differences in oxidative stress and inflammatory responses (14), males may be more susceptible to oxidative stress induced by mitochondrial dysfunction. Females typically exhibit a stronger inflammatory response, which may negatively impact mitochondrial function. There are gender differences in metabolic complications, with men more likely than women to develop non-alcoholic fatty liver disease as well as cardiovascular disease, whereas women are at substantially increased risk after menopause (15). Thus, women may have better mitochondrial function before menopause due to the protective effects of oestrogen, but postmenopausal women have a rapid decline in mitochondrial function and are at increased risk. Men, on the other hand, may be more susceptible to mitochondria-associated oxidative stress and exhibit earlier or more severe metabolic complications.

The duration of diabetes affects mitochondrial function. Diabetic patients with chronically high blood glucose levels produce more ROS, which attack the mitochondrial membrane, leading to mitochondrial damage and affecting normal mitochondrial function (16). This state of oxidative stress increases with the duration of diabetes. Studies have shown that both the number and function of mitochondria in diabetic patients decline progressively with the duration of the disease (17). As the duration of diabetes increases, intracellular metabolic pathways are disrupted and the role of mitochondria in regulating energy metabolism is diminished, potentially leading to further exacerbation of insulin resistance (18). Diabetes-associated hyperglycaemia and oxidative stress may also lead to damage to mitochondrial DNA (19), which can further impair mitochondrial function and affect the metabolic capacity of cells.

T2DM is strongly associated with obesity, and body mass index (BMI) is an important indicator of the degree of obesity. Individuals with high BMI tend to have an excessive accumulation of adipose tissue, leading to abnormalities in adipose tissue function. Such abnormalities include increased oxidative stress, enhanced inflammatory responses, and metabolic disturbances. These factors can damage mitochondria (20), leading to decreased mitochondrial respiration, decreased ATP production, and even mitochondrial DNA damage. High BMI is often accompanied by insulin resistance, which increases metabolic stress, which in turn affects the metabolic flexibility of mitochondria (21) and impairs their ability to oxidise fatty acids. This may further exacerbate disturbances in glycolipid metabolism and affect glucose homeostasis. In conclusion, elevated BMI is closely associated with mitochondrial dysfunction, which plays an important role in the pathogenesis of T2DM. By improving BMI, mitochondrial function can be repaired to a certain extent, thus contributing to the management and prevention of diabetes.

After conducting a thorough search of the pertinent literature, we discovered a significant amount of material exploring the connection between mitochondria and T2DM. However, a systematic and exhaustive visual analysis had not been previously undertaken. Bibliometrics, a quantitative approach, aids in depicting and evaluating the development and advancements within a specific discipline or research area. This study employs both CiteSpace and VOSviewer to objectively outline the knowledge foundation and evolving trends concerning the mitochondria-T2DM relationship. Furthermore, it delves into the primary research focal points and monitors prevalent research topics. Leveraging the insights gained from analyzing the data presented in the literature, this paper strives to offer guidance for future investigations into the mitochondria-T2DM connection and preventative strategies for individuals with T2DM.

## 2 Materials and methods

### 2.1 Data collection

**Figure 1:**
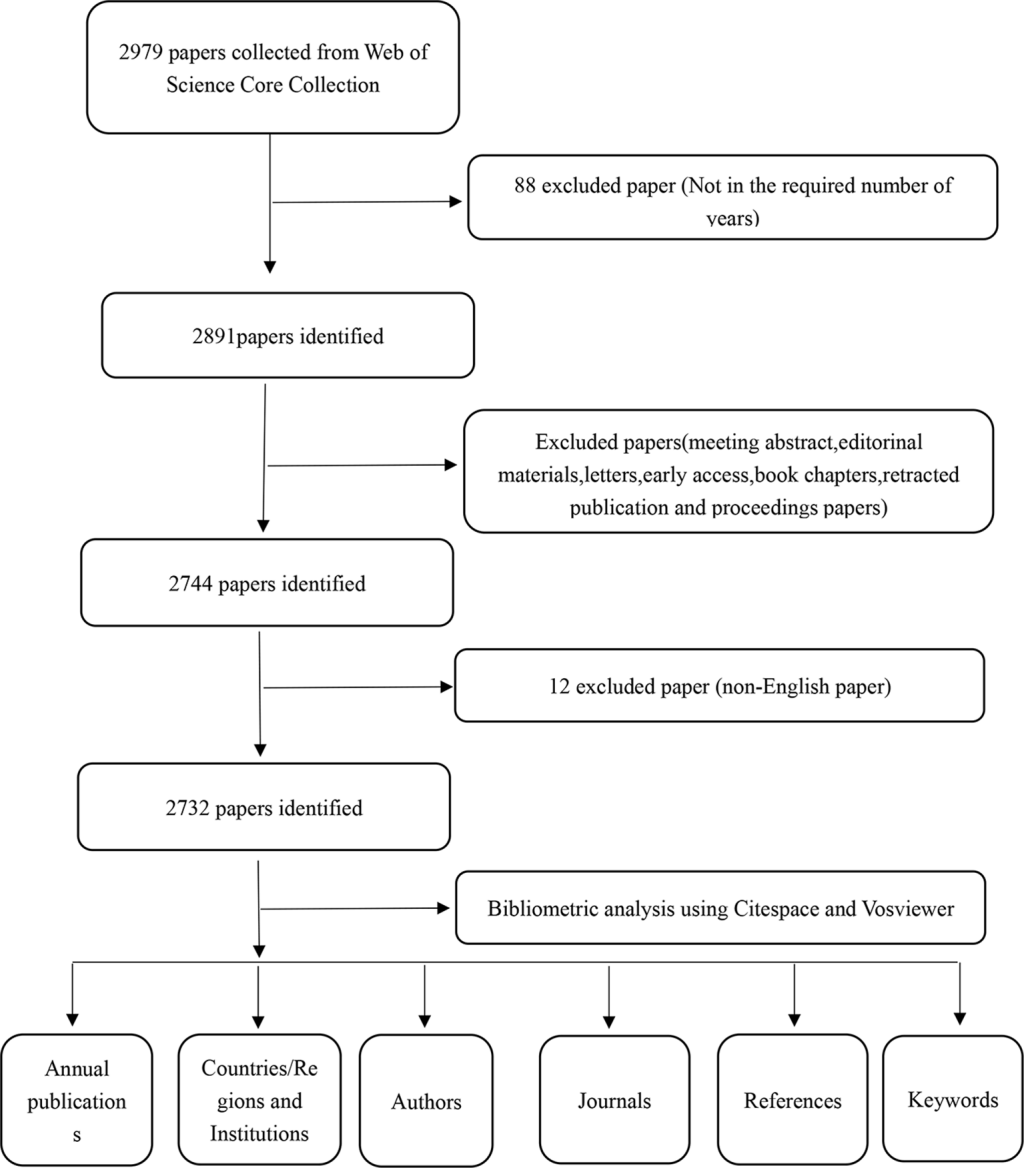
The figure presents a one-frame flow chart that illustrates the comprehensive criteria and the step-by-step process of conducting a bibliometric analysis on publications chosen from the WOS database, specifically exploring the Mitochondria and T2DM.

On July 19, 2024, a thorough search was conducted on the Web of Science Core Collection (WoSCC) using the following search terms: TS=(“mitochondria”OR “mitochondrial contraction”OR”mitochondrial property”)AND TS=(“Type 2 Diabetes Mellitus*” OR”Type 2 Diabetes*”). The search was limited to articles and reviews written in English, excluding conference abstracts, preemptive experience articles, editorial materials, letters, collections, thesis collections, news projects, book chapters, hardware reviews, and withdrawn publications. The retrieval period spans from January 1st, 2003 to December 31st, 2023, encompassing a total of 2732 articles. This selection comprises 2029 articles and 703 reviews. A comprehensive extraction was conducted for each publication, including the title, year of publication, country or region, institution, author, journal, references, and key words. For further insights into the literature extraction process, refer to **Figure 1**.

### 2.2 Data analysis

For bibliometric and knowledge mapping analysis, we utilized CiteSpace 6.3r1, vosviewer 1.6.18, bibliometrix in RStudio (v.4.4.1), and Microsoft Excel 2021.

CiteSpace is a software designed by Professor Chao Mei Chen from Drexel University in Philadelphia, Pennsylvania, USA, specifically to provide a visual representation of knowledge domains (22, 23). Please adjust the time parameter to span from 2003 to 2023, and configure the parameter threshold as 1. Additionally, Top N (N=25),LRF=2.5,LBY=5,e=1.0. In this paper, we examine the operations of countries, institutions, jointly cited references, reference bursts, keywords, and keyword bursts.

The dataset underwent basic statistical analysis utilizing RStudio (v.4.4.1). The data were organized in the bibliometrix format, while the biblioshiny package facilitated the extraction of various features pertaining to research literature spanning from 2003 to 20223 (24). It comprises the primary information, the foremost pertinent authors, their production over time, and the globally most-cited literature for quantitative analysis.

Vosviewer, which was developed by Eck and Waltman from the Leiden scientific and technological research center in the Netherlands, is a software designed for mapping scientific knowledge. This software has the capability to construct and visualize the relationships between network data, revealing the structure, evolution, and cooperation within the knowledge field. Its standout features include a graphical display and powerful large-scale data analysis abilities. In this article, we explore the implementation of various elements such as the author, co-cited author, journal, and co-cited journal.

This article utilizes Microsoft Excel 2021 to examine yearly publication patterns while simultaneously searching for the impact index of magazines and periodicals in 2023 on Web of Science.

## 3 Results

### 3.1 Relevant publication information

**Figure 2:**
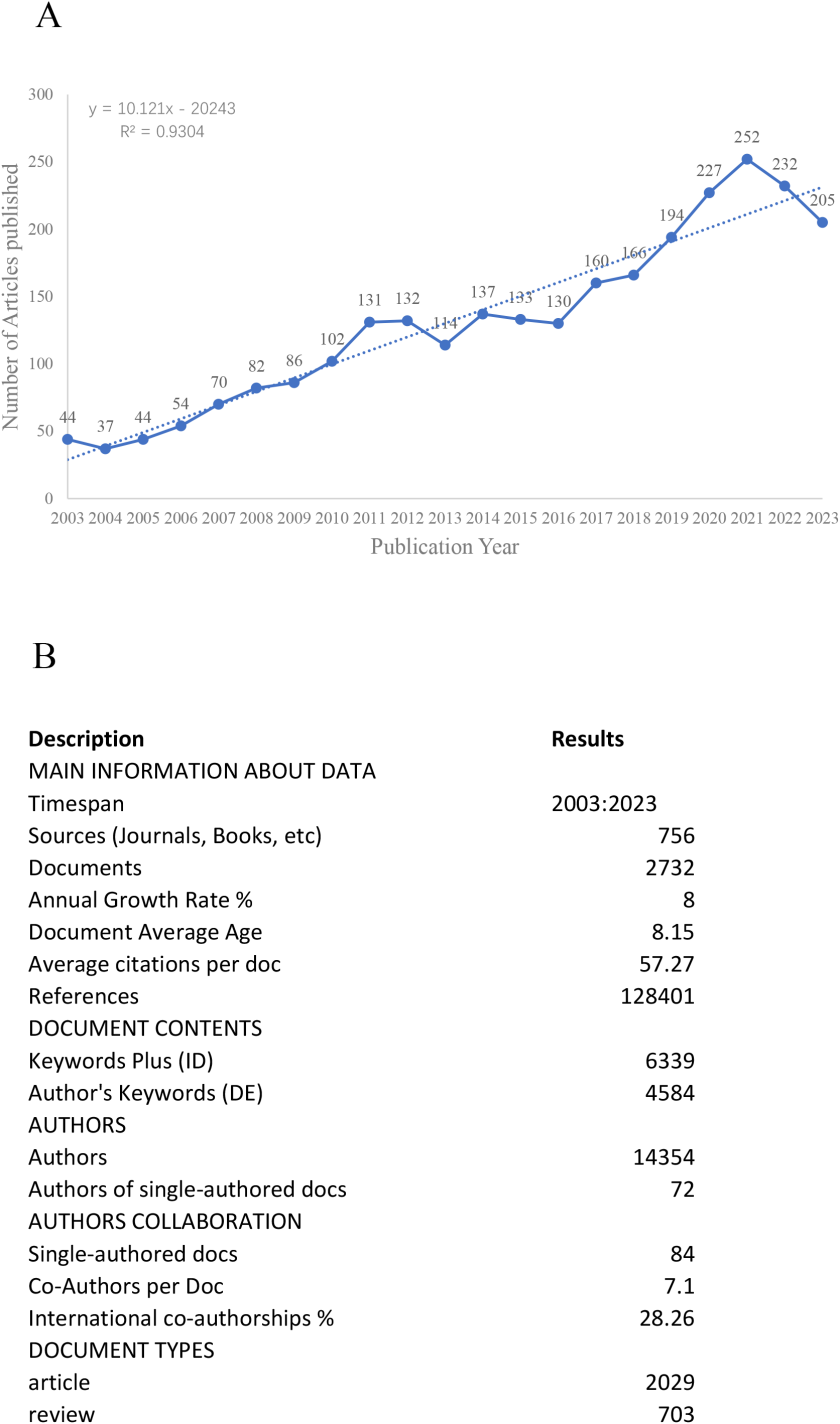
**(A)** Distribution trend from January 1, 2014, to December 31, 2023. **(B)** Basic information about literature.

The objective of this study is to examine 2732 pieces of literature spanning from January 1, 2003, to December 31, 2023, and utilize the collected data to establish the evolving trend of the correlation between mitochondria and T2DM. As illustrated in **Figure 2A**, the number of research articles on this topic steadily rose from 2003 to 2011. Subsequently, from 2011 to 2023, there was a significant surge in publications, reflecting a growing fascination with relevant research on this subject during this timeframe. It is noteworthy that the peak production of 252 articles occurred in 2021. To gain a deeper understanding of the publication trend, a linear trend line for annual publications was created. The resulting equation is y=10.121x-20243, where y denotes annual publications and x represents the year. The model’s coefficient of determination (R²) is 0.9304. **Figure 2B** presents an overview of the analyzed articles, which comprise a total of 128,401 references. The average publication year stands at 8.15. Furthermore, each article has garnered an average of 57.27 citations, and the annual publishing growth rate has been recorded at 8%.

### 3.2 Distribution of countries/regions

**Figure 3:**
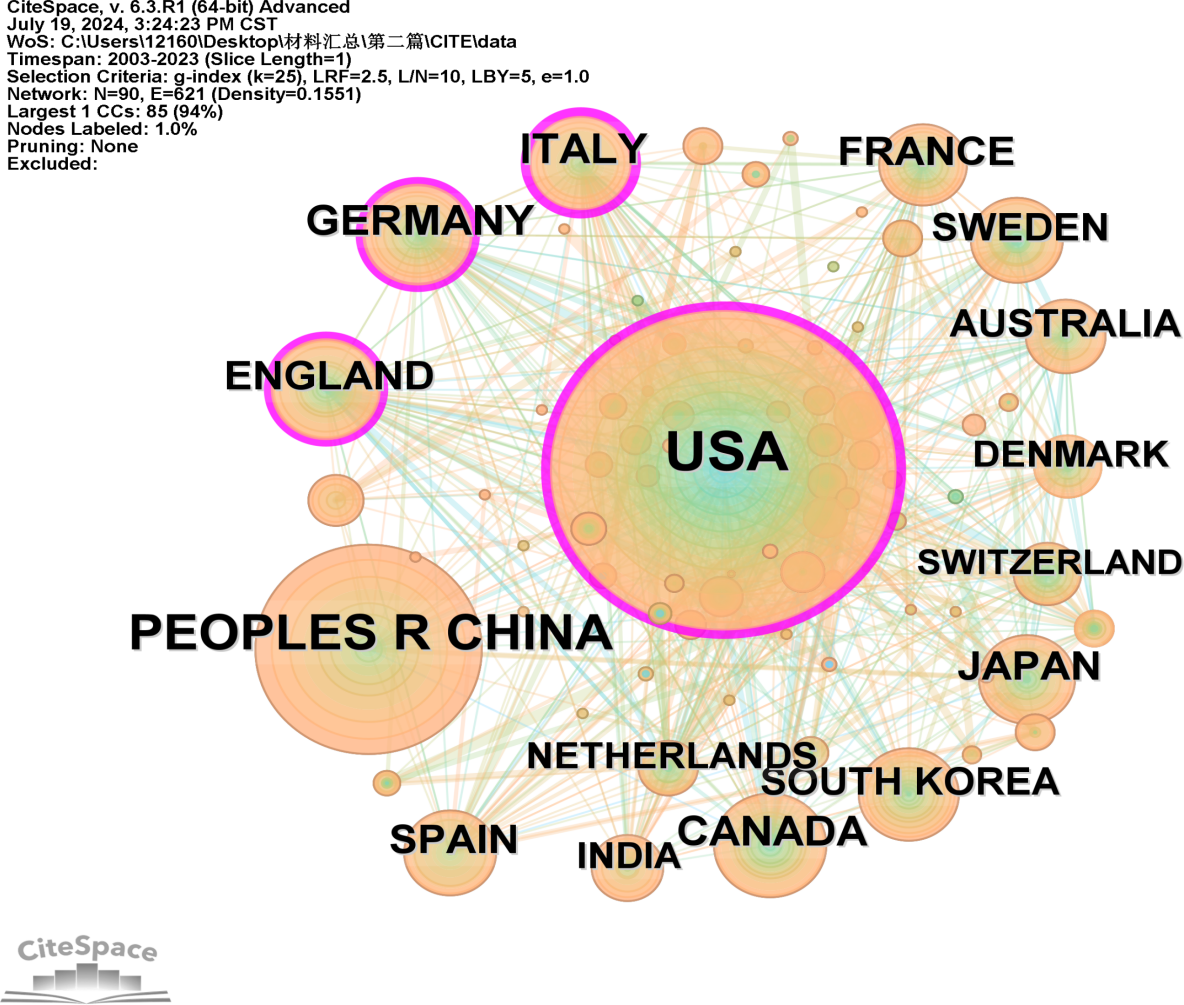
A visualization map of countries.

A total of 493 countries were involved in this study. **Figure 3** illustrates the co-occurrence of countries, with nodes denoting individual countries and the size of each node reflecting the national circulation. Notably, the United States is represented by the largest circle, highlighting its significant influence in the region. The purple segment of the circle symbolizes the center, and the prominent position of the United States suggests frequent collaboration with other nations. Referring to **Table 1**, the top 11 countries with the highest citation frequencies are listed. The United States leads with 988 citations, followed by China with 464 citations, and Italy with 157 citations. In terms of centrality, the United States tops the list with a centrality score of 0.33, Italy ranks second with 0.3, and Germany comes in third with 0.13.

**Table 1:** Top 11 countries/regions and institutions related to Mitochondria and T2DM.

Rank	Country/Region	Count	Centrality	H-index	Citations Per Papers
1	USA	988	0.33	132	72.67
2	CHINA	464	0.07	63	34.18
3	ITALY	157	0.3	51	54.31
4	CANADA	156	0.1	54	74.15
5	GERMANY	154	0.13	51	65.5
6	ENGLAND	142	0.11	49	57.46
7	JAPAN	127	0.04	38	44.46
8	SPAIN	124	0.10	44	67.76
9	FRANCE	118	0.05	46	76.82
10	SOUTH KOREA	109	0.05	41	49.39
	SWEDEN	109	0.04	42	56.16

### 3.3 University and institutional

**Figure 4:**
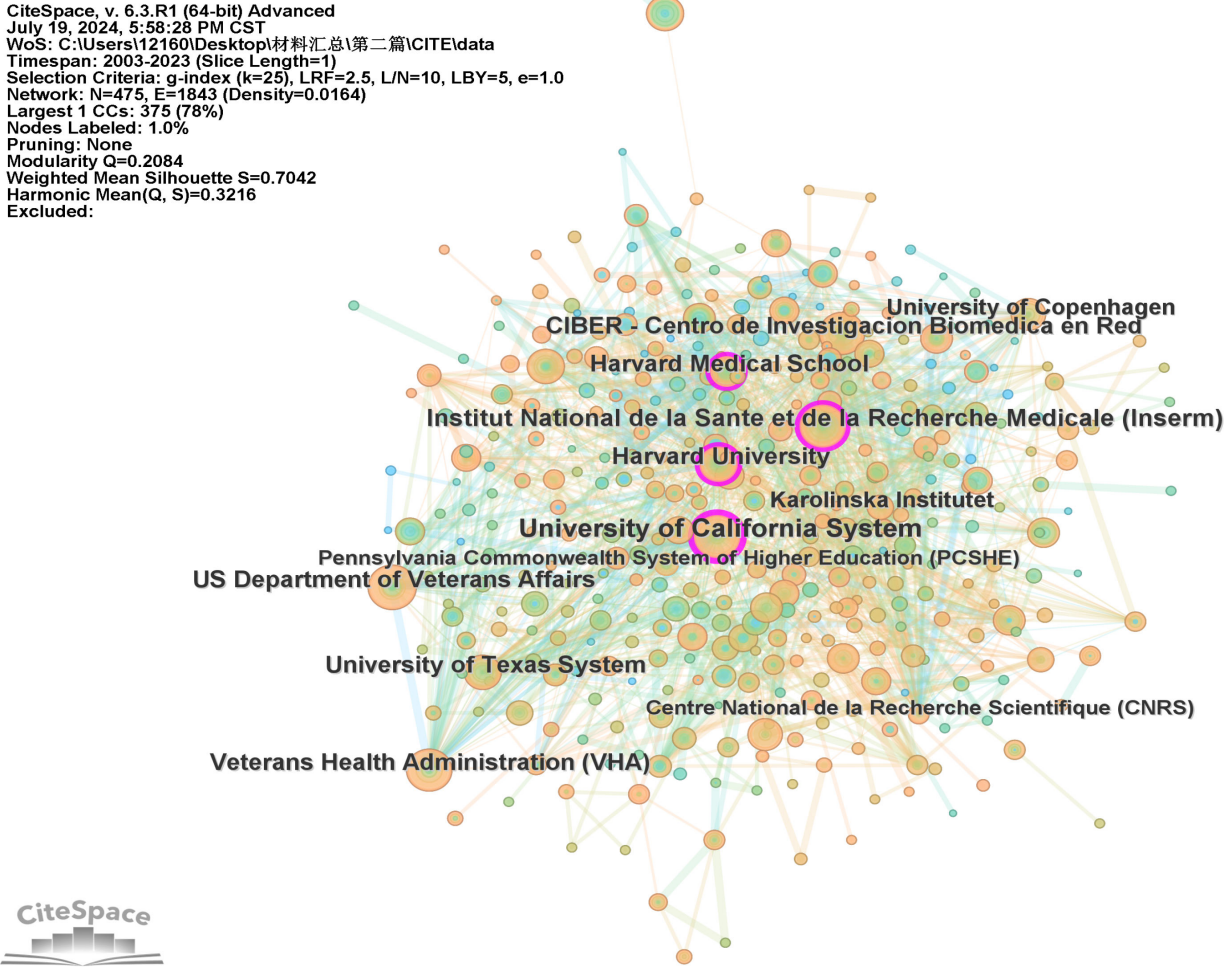
A visualization map of institutions.

A total of 2897 institutions contributed to the research on this subject. The network pruning analysis (**Figure 4**) revealed 475 nodes and 1843 connections in total. **Table 2** presents the top 10 institutions based on their literature output. Notably, the University of California system tops the list with 91 publications, followed by the Institut National de la Santé et de la Recherche Médicale with 81 publications, and the US Department of Veterans Affairs with 67 publications. It is evident from the table that six out of the top 10 institutions hail from the United States, highlighting the country’s profound exploration of this topic and its strong scientific research footing. Furthermore, it’s worth mentioning that the Institut National de la Santé et de la Recherche Médicale demonstrated a higher h-index, signifying its significant impact in publishing scholarly articles.

**Table 2:** Top 10 institutions related to Mitochondria and T2DM.

Rank	Institutions	Counts	H-Index	Countries or Regions
1	UNIVERSITY OF CALIFORNIA SYSTEM	92	41	America
2	INSTITUT NATIONAL DE LA SANTE ET DE LA RECHERCHE MEDICAL INSERM	83	44	France
3	US DEPARTMENT OF VETERANS AFFAIRS	68	33	America
4	HARVARD UNIVERSITY	66	32	America
5	VETERANS HEALTH ADMINISTRATION VHA	65	33	America
6	CIBER CENTRO DE INVESTIGACION BIOMEDICA EN RED	65	40	Spanish
7	UNIVERSITY OF TEXAS SYSTEM	59	35	America
8	HARVARD MEDICAL SCHOOL	55	36	America
9	UNIVERSITY OF COPENHAGEN	49	27	Denmark
10	KAROLINSKA INSTITUTET	44	25	Sweden

### 3.4 Authors and co-cited authors

**Figure 5:**
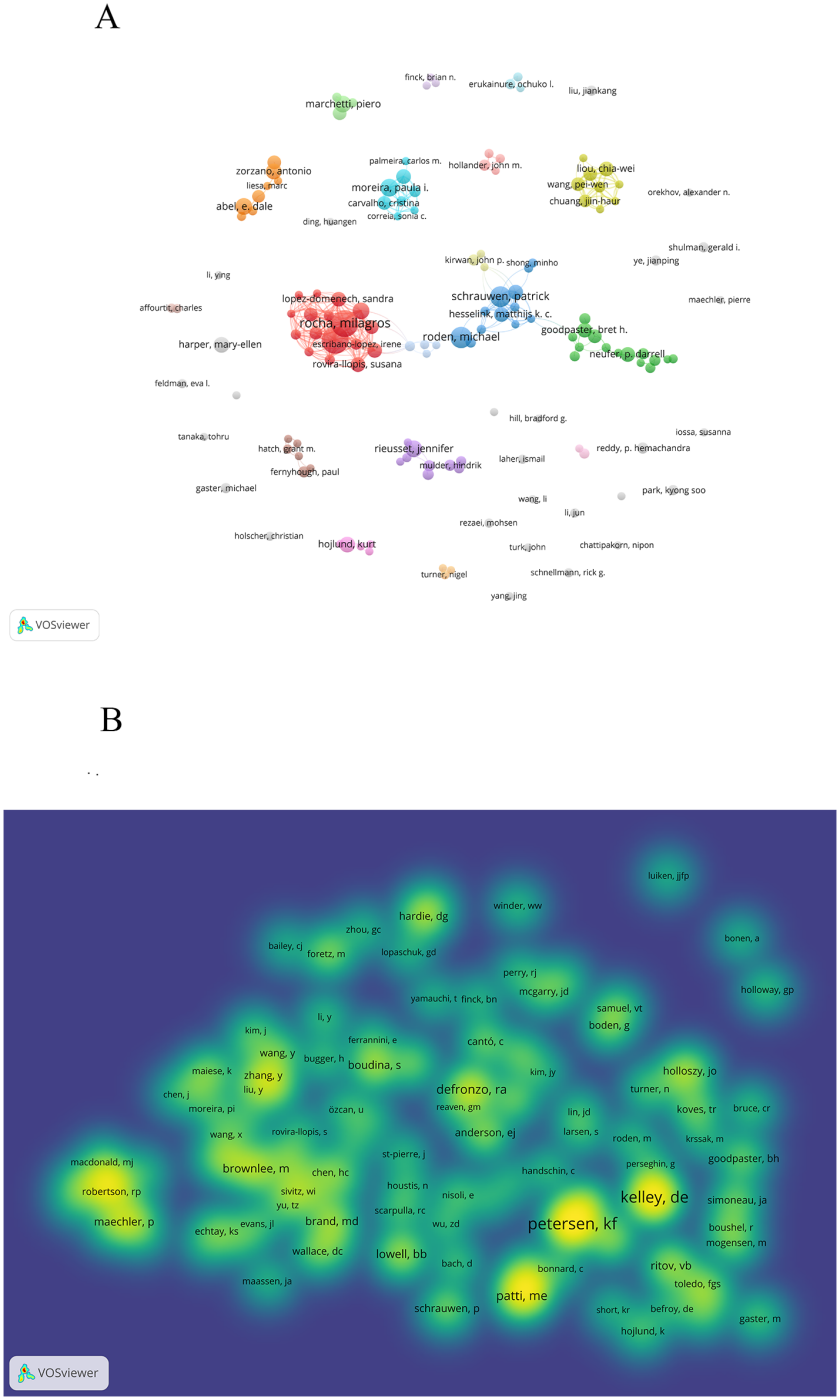
A visualization map of Authors and co-cited authors. **(A)** illustrates the collaboration among the top ten authors. **(B)** demonstrates the aggregation of co-cited authors.

A total of 15,379 authors contributed to the study of this topic, with 29 authors publishing 10 or more articles. **Table 3** highlights the top 10 most prolific authors. Notably, Rocha Milagros,Victor Victor M led the pack, each with 24 articles. Roden Michael (n=18) and Schrauwen Patrick (n=17) closely followed. **Figure 5A** illustrates the co-occurrence of authors, displaying 25 clusters. Within one particular cluster, cooperation is notably more pronounced, However, among the authors actively engaged in this topic, only Rocha Milagros and Banuls Celiaform a distinct cluster. Simultaneously, Roden Michael,Schrauwen Patrick form a closely knit cluster while the remaining authors exhibit less cooperation.

**Table 3:** Top 10 authors and co-cited authors related to Mitochondria and T2DM.

Rank	Author	Count	Co-cited author	Co-citation
1	rocha, milagros	24	petersen, kf	556
	victor, victor m	24	kelley, de	544
3	roden, michael	18	patti, me	289
4	schrauwen, patrick	17	defronzo, ra	274
5	moreira, paula i	14	mootha, vk	256
6	abel, e. dale	13	brownlee, m	228
7	banuls, celia	13	lowell, bb	218
8	marchetti, piero	13	boudina, s	206
9	rieusset, jennifer	13	ritov, vb	201
10	harper, mary-ellen	12	morino, k	194
	hojlund, kurt	12		
	zorzano, antonio	12		

Out of the 78,634 co-cited authors, 48 were co-cited over 100 times, as illustrated in **Figure 5B**. This figure displays them in a density map format, effectively highlighting the high-frequency co-cited authors. The brightness of the color corresponds to the number of citations; the brighter the color, the more citations. Based on **Table 3**, **Figure 5B**, Petersen KF, Kelley De, Patti ME share the most frequent citations.

### 3.5 Journals and co-cited journals

**Figure 6:**
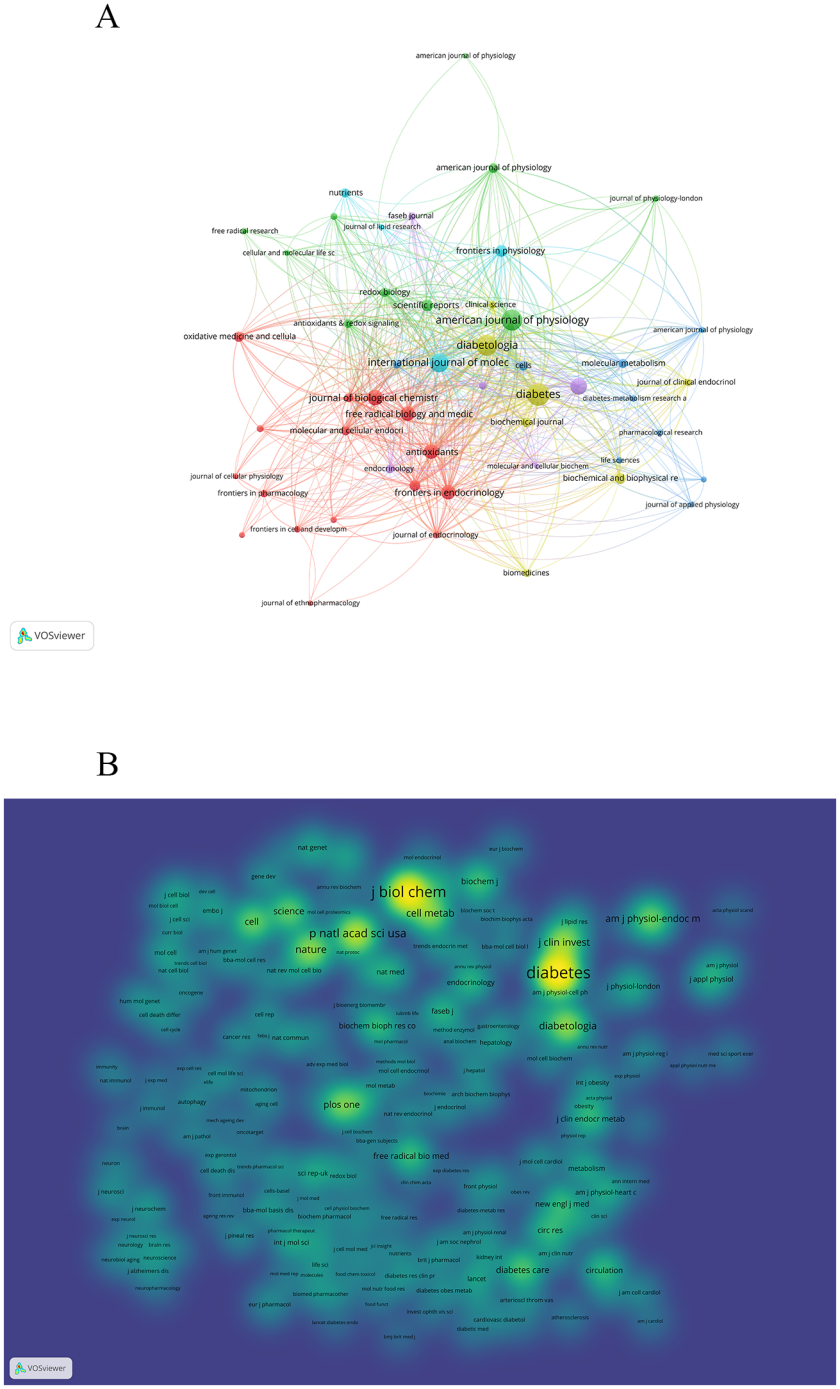
A visualization map of Journal and co-cited journal. **(A)** illustrates the collaboration among the top ten journal. **(B)** demonstrates the aggregation of co-cited journal.

A total of 757 journals have published literature pertaining to this topic. **Table 4**, **Figure 6A** present the top 10 journals with the highest number of published papers. Collectively, these top 10 journals published 540 papers, which constitute 31.17% of the overall published literature. The majority of the papers were published in the DIABETES (n = 80). AMERICAN JOURNAL OF PHYSIOLOGY-ENDOCRINOLOGY AND METABOLISM,DIABETOLOGIA tied for second place with 72 publications each. Notably, DIABETOLOGIA boasts the highest impact factor of 8.4, while the JOURNAL OF BIOLOGICAL CHEMISTRY leads with the highest h-index of 477.

**Table 4:** Top 10 journals related to Mitochondria and T2DM.

Rank	Journal	Count	IF (2023)#	JCR (2023)	H-Index	Country
1	DIABETES	80	6.2	Q1	304	USA
2	AMERICAN JOURNAL OF PHYSIOLOGY-ENDOCRINOLOGY AND METABOLISM	72	4.2	Q1	182	USA
3	DIABETOLOGIA	72	8.4	Q1	207	GERMANY
4	INTERNATIONAL JOURNAL OF MOLECULAR SCIENCES	62	4.9	Q2	114	USA
5	PLoS One	51	2.9	Q2	268	USA
6	JOURNAL OF BIOLOGICAL CHEMISTRY	47	4	Q2	477	USA
7	FREE RADICAL BIOLOGY AND MEDICINE	42	7.1	Q1	239	USA
8	Frontiers in Endocrinology	42	3.9	Q2	51	USA
9	Antioxidants	41	6	Q1		SWITZERLAND
10	Frontiers in Physiology	31	3.2	Q2	75	SWITZERLAND

A total of 8232 journals were cited in research on this topic. Out of these, 31 journals had over 1000 citations each (refer to **Figure 6B**). According to **Table 5**, Diabetes tops the list with the highest number of citations (n=10566), closely followed by JOURNAL OF BIOLOGICAL CHEMISTRY (n=8882) and PROCEEDINGS OF THE NATIONAL ACADEMY OF SCIENCES OF THE UNITED STATES OF AMERICA(n=4838). Within the top 10 co-cited journals, NATURE boasts the highest impact factor at 50.5, preceded by CELL at 45.5 and Cell Metabolism at 27.7. Notably, eight out of the top 10 frequently cited journals are positioned in Q1 of JCR, while the remaining two are in Q2.

**Table 5:** Top 10 co-cited journals related to Mitochondria and T2DM.

Rank	Co-Journal	Count	IF (2023)#	JCR (2023)	Country
1	DIABETES	10566	6.2	Q1	USA
2	JOURNAL OF BIOLOGICAL CHEMISTRY	8882	4	Q2	USA
3	PROCEEDINGS OF THE NATIONAL ACADEMY OF SCIENCES OF THE UNITED STATES OF AMERICA	4838	9.4	Q1	USA
4	JOURNAL OF CLINICAL INVESTIGATION	3862	13.3	Q1	USA
5	DIABETOLOGIA	3845	8.4	Q1	GERMANY
6	NATURE	3835	50.5	Q1	ENGLAND
7	Cell Metabolism	3491	27.7	Q1	USA
8	AMERICAN JOURNAL OF PHYSIOLOGY-ENDOCRINOLOGY AND METABOLISM	3023	4.2	Q1	USA
9	PLoS One	2875	2.9	Q2	USA
10	CELL	2704	45.5	Q1	USA

### 3.6 Co-citations references and references burst

**Figure 7:**
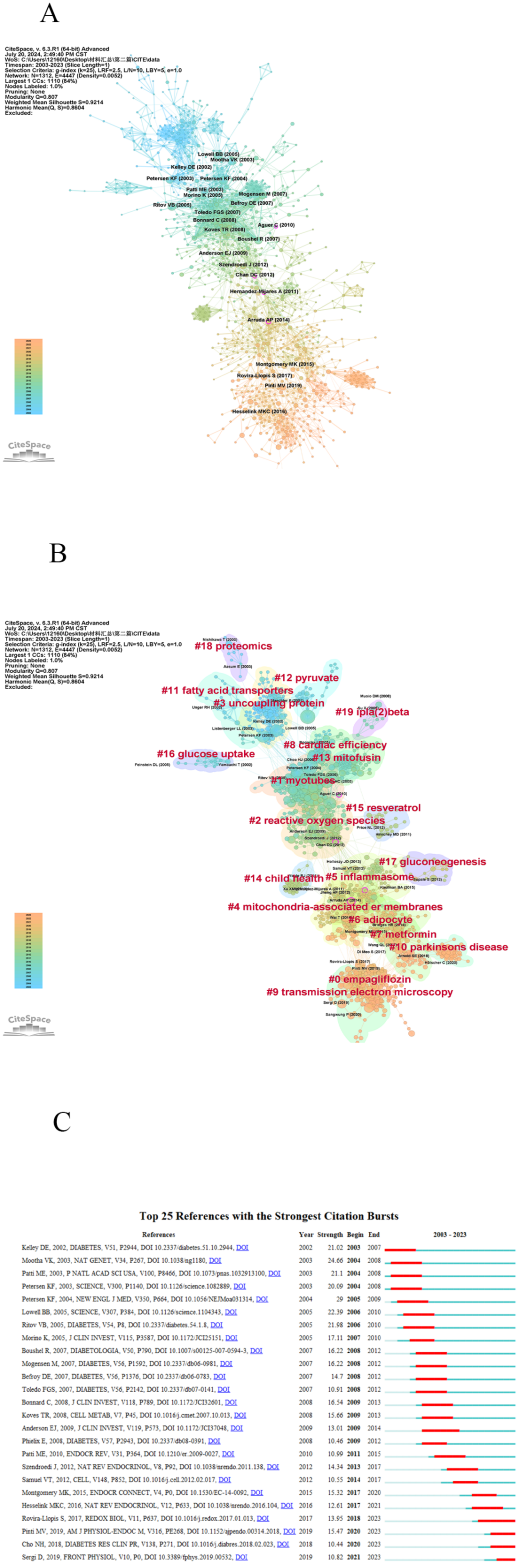
A visualization map of Co-citations references. **(A)** A visualization map of Co-citations references. **(B)** CiteSpace visualization of Cluster analysis of Co-citations references related to Mitochondria and T2DM. **(C)** Top 25 references with the strongest citation bursts involved in Mitochondria and T2DM.

We utilized CiteSpace to assess the influence of highly-cited literature in this research domain. We segmented the data spanning from 2003 to 2023 and extracted the co-cited reference network, which comprises 1312 references and 4447 connections. **Figure 7A**’s left side displays the co-occurrence diagram of co-cited references, where each node signifies a document. The circle’s size indicates the frequency of the journal’s citations, while a purple halo denotes journals with stronger centrality (>0.1). The linkage between nodes signifies collaboration between two journals within a thematic cluster for this research. **Table 6** presents the top 10 co-cited references. Based on the citation count, the most highly ranked reference is “Important mitochondrial activity in the insulin resistant offspring of patients with type 2 diabetes” (25), garnering 64 citations. The second most cited reference is “Mitochondrial dysfunction and type 2 diabetes” (26), with 55 citations, followed by “Deficiency of subsarcolemmal mitochondria in obesity and type 2 diabetes” (27) with 54 citations, rounding out the top three. To obtain the reference clustering analysis diagram (**Figure 7B**), please follow these steps: first, adjust the scale factor by increasing or decreasing it to k=12. Next, select “Pathfinder” to prune the merged network. After that, click on “find cluster,” then select “K,” and finally “LLR.” Through this analysis, 22 clusters are identified, and the modular value (Q value) is determined to be 0.807. This Q value suggests that each cluster possesses distinct and clearly defined characteristics. The average contour value of 0.9214 suggests that the literature exhibits a remarkable homogeneity, which underscores the scholars’ profound exploration and focused concentration in the research domain of this subject matter. The cluster number is marked in the form of increasing from 0. The smaller the number is, the more literatures are included in the corresponding cluster. Among them, “#0 empagliffozin”is the most important cluster, indicating that a large number of studies on “empagliffozin”cite the literatures in the cluster.

**Table 6:** Top 10 Co-citations references related to Mitochondria and T2DM.

Rank	Label	Title	Co-citation	Centrality
1	Petersen KF (2004) (25)	Impaired mitochondrial activity in the insulin-resistant offspring of patients with type 2 diabetes	64	0.02
2	Lowell BB (2005) (26)	Mitochondrial dysfunction and type 2 diabetes	55	0.07
3	Ritov VB (2005) (27)	Deficiency of subsarcolemmal mitochondria in obesity and type 2 diabetes	54	0.06
4	Mootha VK (2003) (28)	PGC-1alpha-responsive genes involved in oxidative phosphorylation are coordinately downregulated in human diabetes	49	0.01
5	Koves TR (2008) (30)	Mitochondrial overload and incomplete fatty acid oxidation contribute to skeletal muscle insulin resistance	44	0.06
6	Boushel R (2007) (31)	Patients with type 2 diabetes have normal mitochondrial function in skeletal muscle	43	0.05
7	Mogensen M (2007) (32)	Mitochondrial respiration is decreased in skeletal muscle of patients with type 2 diabetes	43	0.08
8	Patti ME (2003) (33)	Coordinated reduction of genes of oxidative metabolism in humans with insulin resistance and diabetes: Potential role of PGC1 and NRF1	42	0.01
9	Bonnard C (2008) (34)	Mitochondrial dysfunction results from oxidative stress in the skeletal muscle of diet-induced insulin-resistant mice	41	0.04
10	Petersen KF (2003) (35)	Mitochondrial dysfunction in the elderly: possible role in insulin resistance	40	0.05

CiteSpace has identified the top 25 references experiencing the most significant citation bursts, as illustrated in **Figure 7C**. Notably, the majority of citation outbreaks took place in 2007(4/25,16%), closely followed by 2003(3/25,12%),2005(3/25,12%)and 2008(3/25,12%). Furthermore, it’s remarkable that the citation explosion persisted until 2023 for 4 references(16%).Among these, the article authored by Mootha, VK (28) exhibited the strongest citation burst, with an intensity of 24.66, spanning from 2004 to 2008.

### 3.7 Keywords and Keywords burst

**Figure 8:**
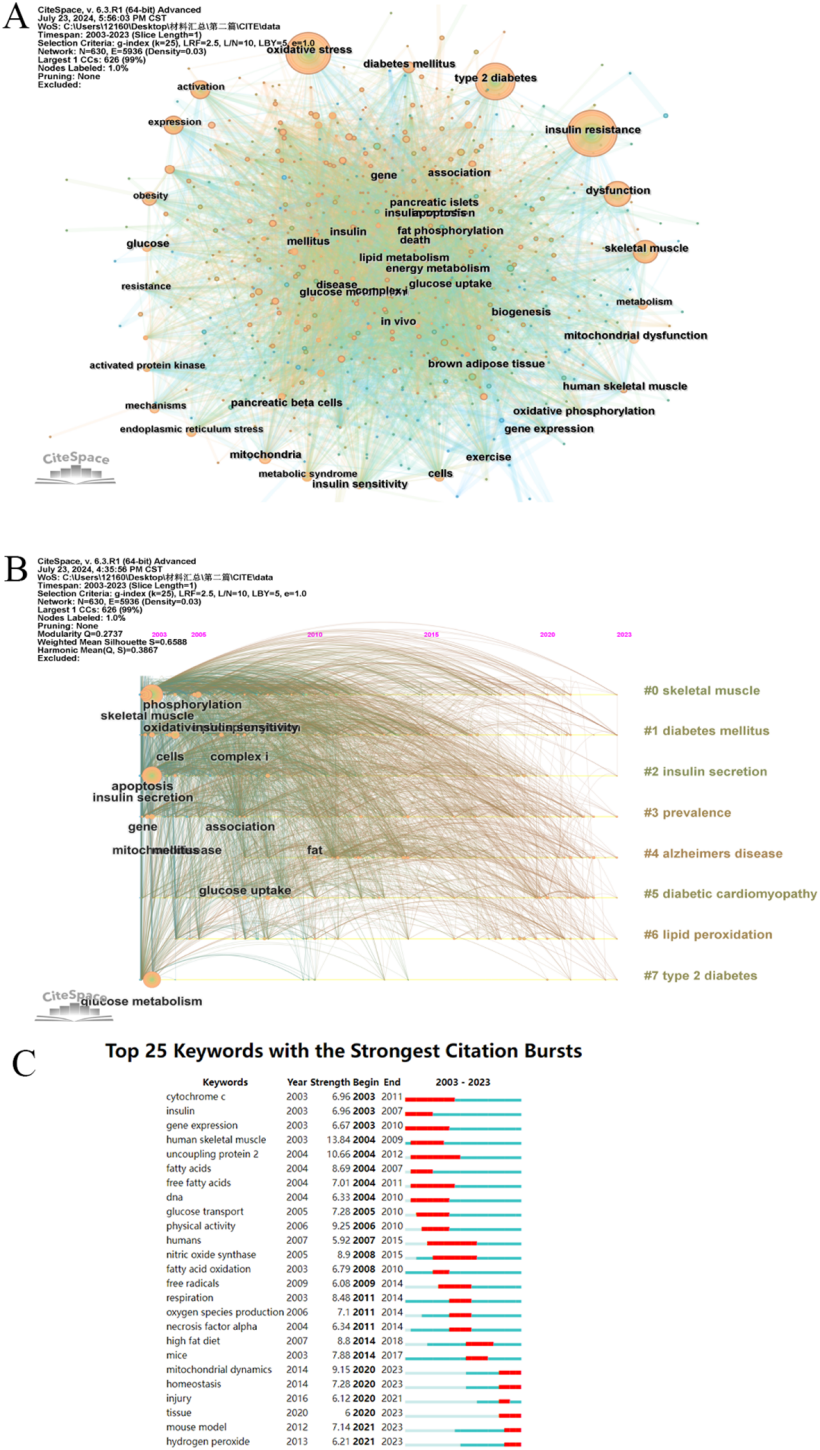
A visualization map of keywords. **(A)** A visualization map of keywords. **(B)** CiteSpace visualization timeline view of keywords clustering analysis related to Mitochondria and T2DM. **(C)** Top 25 keywords with the strongest citation bursts involved in Mitochondria and T2DM.

We employed CiteSpace to create a visual representation of the frequently occurring keywords in the literature pertaining to our study topic. The resulting visualization, depicted in **Figure 8A**, illustrates that the size of the circle corresponds to the frequency of the associated keyword in the literature; a larger circle indicates a higher frequency. Specifically, 209 keywords occurred more than 10 times, while 29 keywords surfaced over 100 times. **Table 7** presents the top ten most popular keywords ranked by their frequency of occurrence. Notably, “oxidative stress”, “insulin resistance”, and “type 2 diabetes”emerge as the highest-ranked keywords. Although high-frequency keywords may be prevalent, not all of them exhibit high centrality. Consequently, solely relying on such keywords may not precisely pinpoint research hotspots. Within the CiteSpace software, keywords boasting a centrality of 0.01 or greater stand out as inflection points on the keyword frequency knowledge map, thereby serving as indicators of research hotspots in the respective field. From the centrality perspective, “energy metabolism”holds a centrality of 0.06, while “apoptosis”and “insulin sensitivity”both possess a centrality of 0.05. These factors serve as the backbone of our research and occupy a supporting position within the network. **Figure 8B** offers a chronological description of keyword clustering analysis, presenting a timeline perspective. The various clusters are represented by brightly colored horizontal lines on the right, and each cluster corresponds to a specific set of keywords. The nodes positioned along these horizontal lines symbolize the keywords themselves. Notably, the spatial distribution of these nodes along the horizontal axis signifies the year in which the respective keywords first emerged in academic literature, ultimately forming a comprehensive temporal portrayal of the evolution of keyword clusters. “0#skeletal muscle”is the largest, followed by “#1diabetes mellitus”, “#2insulin secretion”, “#3prevalence”, “#4alzheimers disease” and”#5 diabetic cardiomyopathy”.

**Table 7:** Top 10 keywords of Mitochondria and T2DM.

Rank	Keywords	Counts
1	oxidative stress	722
2	insulin resistance	713
3	type 2 diabetes	592
4	skeletal muscle	424
5	dysfunction	357
6	expression	282
7	mitochondria	258
8	metabolism	249
9	activation	213
10	obesity	204

The term “keyword burst”denotes the abrupt surge of specific keywords within associated domains during a particular timeframe. **Figure 8C** illustrates the top 25 keywords experiencing the most significant citation explosions. Among them, “human skeletal muscle”exhibits the highest outbreak intensity, closely followed by “uncoupling protein 2” and “physical activity”. It is noteworthy that “mitochondrial dynamics”, “homeostasis”, “injury”, “tissue”, “mouse model”, and “hydroperoxide”were all in a state of explosion until 2023, with “uncoupling protein 2” emerging as the most prominent factor.

## 4 Discussion

### 4.1 General information discussion

In this study, a scientometric analysis was conducted on the scientific literature pertaining to mitochondria and T2DM in the Web of Science (WOS) database, spanning from 2003 to 2023. The analysis was carried out using CiteSpace, VOSviewer, and Bibliometrix, with the aim of exploring the relationship between mitochondria and T2DM in the scientific literature.

From 2003 to 2023, a total of 2732 articles were included. The publication trends of these articles over time reflect the evolving interest in this research field. Initially, from 2003 to 2011, the number of papers grew gradually, but there was a significant surge in publications from 2011 to 2023, indicating a marked increase in attention and interest in this topic.The distribution of papers across different countries reveals each nation’s scientific impact in this field. Notably, the United States, China, and Italy are leading contributors, collectively accounting for 58.89% of all published papers. The United States stands out with the highest centrality in this research area, demonstrating extensive collaboration with other countries. This suggests that the US has conducted substantial and influential research in this domain.Given this, research teams from Asian countries might consider enhancing their international collaboration, particularly with European and American counterparts, to broaden their impact. It’s worth noting that among the top 10 institutions contributing to this field, 60% are American, and all belong to developed countries. This underscores the depth and thoroughness of research in these nations compared to developing countries, which have a later start and need to enhance their research outputs. While most institutions prefer domestic collaborations, international cooperation remains relatively limited.

Following a thorough examination of the author network, I have identified Rocha Milagros, Victor Victor m, Roden Michael, Schrauwen Patrick, and Moreira Paula as ranking within the top five based on the total number of publications. Therefore, researchers and teams engaged in pertinent studies should prioritize collaborating and communicating with these individuals for future research endeavors. It is noteworthy that Rocha milagros and Victor Victor m published a review in 2017 (29) that delved into the molecular pathways of mitochondrial dynamics, their damage caused by T2DM, and the pathophysiological importance of compromised mitochondrial dynamics in T2DM. This review has been cited 221 times. Petersen KF, Kelley De, Patti ME, Efronzo RA, and Mootha VK are ranked within the top 5 based on the number of co-authors. This suggests that these individuals have established themselves as cornerstone figures in this research field and have made substantial contributions to the advancement of the topic.

The analysis of journals and co-cited journals revealed that Diabetes had published the highest number of studies on mitochondria and T2DM, and had garnered the most co-citations. The AMERICAN JOURNAL OF PHYSIOLOGY-ENDOCRINOLOGY AND METABOLISM, PLoS One, and JOURNAL OF BIOLOGICAL CHEMISTRY are ranked among the top 10 published journals, as well as being the top 10 co-cited journals. This underscores their significant role in the research of this subject matter. If journals like CELL, CELL Metabolism, and Nature are ranked among the top 10, the papers they publish can offer a solid theoretical foundation for future research.

### 4.2 Knowledge base analysis

The Co-citations references can demonstrate the research foundation pertaining to this subject and offer substantial reference value for future investigations on this topic. In our study, we employed CiteSpace to examine the Co-citations references, and the top 10 most frequently cited works are listed below.

In 2003, the New England Journal of Medicine published the highly cited research (25), co-authored by KITT Falk Petersen and five other esteemed scholars. This study compared the age, height, weight, and physical activity of the offspring of healthy, young, thin individuals with those of offspring from patients with insulin-resistant T2DM and IR control subjects. Through an assessment of liver and muscle insulin sensitivity, intramuscular lipid and triglyceride content in muscle cells, whole-body and subcutaneous lipolysis incidence, as well as mitochondrial oxidative phosphorylation activity in muscle, it was determined that a genetic defect in mitochondrial oxidative phosphorylation among T2DM patients results in dysregulation of skeletal muscle IR and intramuscular fatty acid metabolism in the offspring with IR. Additionally, this study underscores mitochondrial oxidative phosphorylation as a promising target for the prevention and treatment of T2DM.

The second most frequently co-citations references study is the one published by Bradford B Lowell in Science in 2005, titled “mitochondrial dysfunction and type 2 diabetes” (26). The maintenance of normal blood glucose levels relies on a complex interplay between the insulin responsiveness of skeletal muscle and liver, and the insulin secreted by pancreatic β cells upon glucose stimulation. Defects in the former lead to IR, while impairments in the latter result in the progression to hyperglycemia. Our study verifies that mitochondrial dysfunction underlies these two distinct characteristics of T2DM.

The third co-citations references is a study published in diabetes by Vladimir B Ritov et al. in 2005 (27). This study aimed to explore the correlation between the subcellular distribution of mitochondria in human skeletal muscle and IR in T2DM and obesity. To achieve this, mitochondrial components of myofibrils were observed after conducting a percutaneous biopsy of the vastus lateralis muscle. The subjects included 11 T2DM patients, 12 age-, gender-, and weight-matched obese sedentary nondiabetic volunteers, and 8 healthy volunteers.

In 2003, vamsi K Mootha and his team published their fourth research in NAT Genet, which was co- citations as reference (28). In this study, gene set enrichment analysis was employed to pinpoint a cluster of genes linked to oxidative phosphorylation. Notably, the expression of these genes is uniformly downregulated in human diabetic muscle. Conversely, their expression is elevated at the site where insulin facilitates glucose disposal, a process activated by PGC-1 α and correlated with overall aerobic capacity.

The fifth most frequently co-citations references is a study collaboratively conducted by Timothy R Koves and his team, which was published in Cell Metabolism in 2008 (30). Utilizing targeted metabolomics, this article verifies that obesity-associated IR in skeletal muscle is marked by excessive β-oxidation, compromised conversion to carbohydrate substrates during the fasting-to-eating transition, and concurrent depletion of organic acid intermediates within the tricarboxylic acid cycle. These findings underscore a profound connection between skeletal muscle IR and lipid-induced mitochondrial stress.

“Patients with type 2 diabetes have normal mitochondrial function in skeletal muscle” (31), authored by R Boushel et al. and published in Diabetologia in 2007, ranks as the sixth most frequently cited reference. In this study, a high-resolution breath assay was employed to measure the oxidative phosphorylation and electron transport capacity of permeabilized muscle fibers from biopsy samples of the quadriceps femoris muscle taken from both healthy subjects and patients with T2DM. The aim was to verify whether the mitochondrial function of T2DM patients was unaffected, and to ascertain if the coupled and uncoupled respiratory retardation observed could be attributed to a reduced mitochondrial content.

The seventh article is a study on diabetes, authored by Martin Mogensen and his team, and published in a renowned publication (32). This study evaluated 3-hydroxyacyl-CoA-dehydrogenase and citrate synthase activities, uncoupling protein content, oxidative stress (measured by 4-hydroxy-2-nonenal), fiber type distribution, and mitochondrial respiration in muscle biopsies obtained from T2DM patients and non-diabetic male subjects. The findings revealed mitochondrial respiratory dysfunction and an elevated number of 2x type fibers in the muscles of T2DM patients.

In 2003, Mary Elizabeth Patti and her team published a study in Proceedings of the National Academy of Sciences of the United States of America (33). This study analyzed skeletal muscle gene expression in both diabetic and non-diabetic patients, demonstrating the presence of PGC1 α and β, as well as multiple oxidative metabolism genes, in high-risk non-diabetic subjects with diabetes mellitus and a family history of diabetes.

The ninth article, authored by Charlotte Bonnard et al. and published in J Clin Invest in 2008, is an experimental study (34). After prolonged dietary intervention with a high-fat, high-sucrose diet, mice developed a diabetic state. In this condition, alterations in mitochondrial biogenesis, structure, and function were noticed in the mouse muscle tissue. This confirms that mitochondrial dysfunction is not an early occurrence in the progression of IR in diabetic mice, but rather a consequence of reactive oxygen species production in skeletal muscle triggered by hyperglycemia and hyperlipidemia.

The tenth co-citations references, authored by KITT Falk Petersen (35) and his team, was published in Science in 2003. This study has verified that IR in elderly individuals is linked to an elevation of fatty acid metabolites in muscle cells, potentially stemming from the age-related decline in mitochondrial oxidation and phosphorylation activity.

In summary, the initial ten co-citations references collectively emphasize the clinical investigation and data gathering contrasting T2DM patients with non-T2DM patients, particularly exploring mitochondrial function, skeletal muscle mitochondria, and their correlation with T2DM. Liver mitochondrial dysfunction is characterised by disturbances in energy metabolism, fat accumulation and oxidative stress, while skeletal muscle mitochondrial dysfunction is characterised by insulin resistance, abnormal lipid metabolism and reduced exercise capacity. Together, hepatic and skeletal muscle mitochondrial dysfunction contributes to the metabolic imbalance in T2DM. Hepatic mitochondrial dysfunction increases glucose output, whereas skeletal muscle mitochondrial dysfunction decreases glucose uptake, both of which together lead to increased hyperglycaemia and insulin resistance. Meanwhile, abnormal fatty acid metabolism interacts in liver and skeletal muscle. Decreased hepatic mitochondrial fatty acid oxidation capacity increases plasma levels of free fatty acids, which are transported to skeletal muscle, further affecting insulin signalling and energy metabolism in muscle. It is noteworthy that the top ten co-cited references have more discussion around skeletal muscle mitochondrial function.This establishes a groundwork for the ensuing discussion on the connection between T2DM and mitochondria.

### 4.3 Hot topics and introductory discussion

In Bibliometrics, the co-occurrence of keywords can indicate the prevalent topics in an academic field, while bursts in keywords and reference citations can reveal emerging topics within a discipline. This study aims to identify the trending topics and cutting-edge research areas by examining reference clustering, bursts in references and keywords, as well as conducting timeline mapping of keyword clustering.

According to the clustering network diagram of co-citations references, the publication time of #18 proteomics, #11 fatty acid transporters, #12 pyruvate, #3 uncoupling protein, #19 ipla (2) beta, #16 glucose uptake, #8 cardiac efficiency, #13 mitofusin, #1 myotubes is generally far from now, These early research papers are primarily located on the left side of the network. For instance, Nishikawa and colleagues (36) investigated three distinct biochemical pathways involved in vascular endothelial cell injury caused by hyperglycemia. They discovered that specific inhibitors targeting these pathways could restore mitochondrial reactive oxygen species levels to normal, thereby preventing hyperglycemic damage. The publication time of the clustered literatures on the right side of the co-citation network is closer now. Further combining the time characteristics of the co-citation relationship and the average time of various literatures, it can be concluded that the current research fields of mitochondria and T2DM are mainly #4 mitochondrial-associated er membranes, #5 inflammasome, #6 adipocyte, #7 metformin. Ana Paula Arruda and her colleagues (37) discovered that obesity results in a significant reorganization of the mitochondria-associated endoplasmic reticulum in the liver. This reorganization causes mitochondrial calcium overload, diminishes mitochondrial oxidative capacity, and increases oxidative stress. Research frontiers and emerging trends are primarily concentrated in clustering #0 empagliflozin, #9 transmission electron microscope, These studies primarily examined the association between mitochondrial dysfunction and both T2DM and IR (38, 39).

References experiencing significant citation explosions can also be identified by the emergence of new themes within a specific field. Out of the top 25 references burst, 4 studies are currently undergoing a citation explosion. These articles reflect the newest and most prominent research topics related to this subject and indicate potential avenues for future exploration,Examples include mitochondrial dynamics, mitochondrial dysfunction, and diabetes prevalence (29, 38–40). Among the remaining 21 references, 11 studies primarily focused on detailing the role of mitochondria in skeletal muscle and its association with T2DM and IR (27, 28, 30–32, 34, 41–45); Six studies were focused on exploring the impact of mitochondrial dysfunction on T2DM and IR (25, 26, 33, 35, 46, 47); Four studies specifically examined the role of mitochondria in T2DM and IR (48–51).

The keyword analysis reveals a close association between mitochondria and T2DM. Additionally, skeletal muscle mechanisms such as mitochondrial dysfunction, oxidative stress, and energy metabolism, along with other factors, are intricately linked to T2DM and its pathological manifestations like obesity, IR, and insulin sensitivity. The keyword analysis aligns with the results obtained from the sudden analysis of Co-citations references. Furthermore, the timeline graph analysis of keywords provides a detailed illustration of the time span of each cluster and the connections under various labels. In this field, the time span of #0,#1 is relatively large. It shows that the research on skeletal muscle mitochondria in diabetes has been paid attention by relevant researchers.

Emergent words refer to terms whose frequency of usage experiences a sudden surge within a specific timeframe, serving as a distinct indicator of shifting research priorities. Over the past two decades, investigations into this subject matter can be broadly categorized into two primary phases. Between 2003 and 2015, research efforts were predominantly centered around clinical applications. However, since 2015, there has been a notable shift towards terms like mitochondrial dynamics, organization, and mouse models, reflecting a current research emphasis on deeper explorations of underlying mechanisms.

According to the above analysis, we have summarized the following three aspects:

#### 4.3.1 relationship between mitochondria and T2DM

Studies have revealed that diabetic patients experience a decrease in mitochondrial oxidative function in their muscles (52), a reduction in mitochondrial capacity within adipose tissue (53), compromised mitochondrial function in the liver (54), and diminished mitochondrial capacity in beta cells (55). The mitochondrial function in tissues implicated in the pathogenesis of diabetes, namely the liver, muscle, adipose tissue, and pancreatic beta cells, plays a pivotal role in various aspects of cellular metabolism. It is imperative that mitochondrial oxidative activity in each tissue is adequate for the complete oxidative loading of nutrients, particularly fatty acids. Any deficiency in this oxidation process can result in the accumulation of lipid intermediates, incomplete fatty acid oxidation products, and reactive oxygen species (ROS). This, in turn, can trigger insulin resistance (in muscle, liver, and fat) and altered secretion in beta cells (49).

#### 4.3.2 Mitochondrial alterations in skeletal muscle in T2DM

Skeletal muscle, a mitochondrial-rich tissue, heavily relies on oxidative phosphorylation for energy generation. In T2DM, skeletal muscle mitochondria experience impairments in bioenergetic capacity (43), a disproportionate decrease in electron transport chain activity within the submuscular mitochondrial component (27), reduced respiratory capacity per mitochondrial unit (32), genetic defects affecting mitochondrial oxidative phosphorylation activity in muscles (42), compromised mitochondrial function, and inflexible metabolism in skeletal muscles (42). However, mitochondrial dysfunction and lipid accumulation in myocytes may exacerbate IR induced glucose metabolism, ultimately triggering T2DM (56).

#### 4.3.3 Impact of mitochondrial dysfunction on T2DM

Mitochondrial dysfunction encompasses a reduction in mitochondrial content, biogenesis, and the expression of mitochondrial oxidative proteins, specifically those involved in the electron transport chain complexes. These factors can collectively hinder substrate oxidation, disrupting the electron flow through the transport chain. This disruption can cause electrons to leak to oxygen, resulting in the formation of superoxide. Both superoxide and other reactive oxygen species (ROS) have the potential to harm various mitochondrial and cellular components. This includes causing oxidative damage to mitochondrial DNA, protein aggregation, and lipid peroxidation. Under high stress conditions, this damage may trigger mitophagy (a process that eliminates damaged mitochondria to prevent cell death) or apoptosis. The removal of mitochondria via mitophagy can further decrease the number of mitochondria, exacerbate lipid accumulation, and potentially lead to metabolic disorders like T2DM (46).

Mitochondrial dysfunction has also been associated with the development of several diabetic complications, such as diabetic cardiomyopathy, neurodegenerative diseases, and erectile dysfunction (ED).The prevalence of ED in patients with T2DM is significantly higher than that in the healthy population, with a prevalence of more than 65 per cent of ED in patients with T2DM over the age of 40 years (57). In the corpus cavernosum of diabetic penises, increased levels of glucolipid metabolism and oxidative stress lead to excessive mitochondrial autophagy (58). Mitochondrial dysfunction usually causes erectile dysfunction in several ways: endothelial dysfunction: mitochondrial dysfunction leads to increased oxidative stress in the endothelium, impairing nitric oxide (NO) production and release. Whereas NO is a key molecule in promoting penile vasodilation and maintaining erection, insufficient NO leads to impaired vasodilatation, which in turn causes ED (59); impaired smooth muscle function: the normal functioning of penile cavernous smooth muscle is essential for erection, and mitochondrial dysfunction may affect the energy supply to these smooth muscle cells (60), leading to a decrease in their relaxation capacity, which in turn aggravates the severity of ED; Linkage of metabolic and vascular complications: patients with T2DM are often associated with metabolic problems such as hypertension, hyperlipidaemia and atherosclerosis, which exacerbate vascular damage. In turn, mitochondrial dysfunction interacts with these pathological processes (61), worsening vascular endothelium and penile blood flow, further affecting erectile function. Therefore, by improving mitochondrial function, such as reducing oxidative stress and promoting energy metabolism, it is possible that this could be a new avenue for the future treatment of T2DM-associated ED.

## 5 Limitations

This study focused solely on retrieving data from the commonly used WoS Core database, neglecting research from other databases, which may have led to the omission of certain pertinent articles. Furthermore, our search was limited to articles written in English, excluding high-quality non-English studies from our analysis. Thirdly, we only considered articles and reviews, disregarding other political and social publications like editorials and books. fourthly, researcher bias is a possibility, as the screening process of the literature required the artificial exclusion of articles that were not relevant to the study. Finally, this study focused only on clinical practice in mitochondrial T2DM, which may have led to the omission of some potential benefits.

## 6 Conclusion

This study employed various bibliometric analysis software to examine the research landscape of mitochondria and T2DM from multiple perspectives. It comprehensively covers details pertaining to countries, journals, authors, institutions, and scientific categories. Furthermore, it offers insightful explanations on the interconnections between frequently cited references and keywords within distinct clustering groups, and their subsequent influence on the field. Moreover, our findings suggest that to gain a deeper understanding, future research should prioritize topics such as particle dynamics, mitochondrial dysfunction, and mechanistic investigations. This bibliometric analysis stands to provide enriched insights for researchers engaged in this domain.
